# Comparative characterization of biochars produced at three selected pyrolysis temperatures from common woody and herbaceous waste streams

**DOI:** 10.7717/peerj.6784

**Published:** 2019-04-15

**Authors:** Matthew Askeland, Bradley Clarke, Jorge Paz-Ferreiro

**Affiliations:** 1 School of Engineering, RMIT University, Melbourne, Australia; 2 School of Science, RMIT University, Melbourne, Australia

**Keywords:** Biochar, Australia, Pyrolysis, Contaminants, Characterization, Waste

## Abstract

Biochar, the product of biomass pyrolysis, has been explored as a soil amendment and carbon capture vessel. Recent literature has aligned biochar as a novel sorbent for a host of environmental contaminants. Through the variation of pyrolysis conditions, biochars can be engineered to have qualities desirable in sorbents whilst maintaining their agronomic benefits. This study focuses on identifying the effects that feedstock type and process temperature have on biochar characteristics which may in turn shed light on their potential environmental applications. Using this approach, six biochars were created from two waste biomasses. The biochars exhibited wide ranges of pH (5.6–11.1), surface area (16.2–397.4 m^2^/g), electrical conductivity (19–2,826 µS/cm), fixed carbon (72–97%), heavy metal and polycyclic aromatic hydrocarbons (PAHs). Statistically significant trends (*P* < 0.05) in biochar characteristics dependent upon increasing pyrolysis temperature and feedstock type were identified. Arsenic (>13 mg/kg), chromium (>93 mg/kg), copper (>143 mg/kg) and PAH (>6 mg/kg) concentrations presented themselves as obstacles to land application in a small number of biochars with respects to International Biochar Initiative (IBI) guidelines. However, it was demonstrated that these could be eliminated through employing pyrolysis processes which encompass higher temperatures (>500 °C) and ensuring the use of contaminant-free feedstocks. The variation in surface areas, carbonized fractions and surface functional groups achieved suggest that using the correct feedstock and process, biochar could be produced in Victoria (Australia) from common organic waste streams to the ends of acting as a sorbent, soil enhancer, and a waste management strategy.

## Introduction

Biochar is the carbonaceous solid resulting from the thermochemical conversion of biomass in an oxygen-limited environment (International Biochar Initiative, 2013). Waste biomass is the largest and most sustainable biomass source, with 220 billion dry tons being produced globally each year (Azargohar et al., 2013). Application of biochar to soil has been demonstrated to improve soil fertility by increasing cation exchange capacity (CEC), soil organic matter content and nutrient availability of the pre-*Terra pretan* soils (Glaser et al., 2000; Glaser et al., 2001; Heitkötter & Marschner, 2015).

Interest in biochar as a tool for carbon sequestration in soil (Lehmann, 2007) soon developed into a focus on biochar’s agronomic potential (Liu et al., 2013). Incorporation of biochar into biocomposites has expanded biochars applications further into the material sciences (Das & Sarmah, 2015; Das, Sarmah & Bhattacharyya, 2016). Biochar can also be used as a novel material for remediation, where contaminant sorption to biochar surfaces reduces bioavailability and mobility (Paz-Ferreiro et al., 2014; Srinivasan & Sarmah, 2015). Biochar characterisation studies with respects to the effect of feedstock and temperature are imperative for adequate decision making in proceeding towards engineering the biochars of the future (Gascó et al., 2018; Lu et al., 2018).

Biochar is primarily composed of stable aromatic carbon ring structures (Al-Wabel et al., 2013), that impart resistance to degradation by oxidants (Mitchell, Dalley & Helleur, 2013) and biological decay (Al-Wabel et al., 2013; Freddo, Cai & Reid, 2012). Its structure gives biochars an estimated residence time in temperate environments of up to 4000 years (Kuzyakov, Bogomolova & Glaser, 2014). The recalcitrance of biochar in the environment varies greatly and is influenced by pyrolysis method and choice of feedstock. Biochar includes a diverse group of materials, with each exhibiting unique physiochemical characteristics and environmental lifespans (Jouiad et al., 2015; Qian et al., 2015).

Pyrolysis temperature governs porosity of the biochar formed due to degassing of volatiles and fracturing through subsequent cooling and shrinkage (Das & Sarmah, 2015). The number and types of surface functional groups present on biochar are also highly temperature dependent, due to volatility, which can result in loss or transformation at higher temperatures (Das & Sarmah, 2015).

Biochar produced from contaminated feedstocks is likely to be contaminated with heavy metals, or pesticide residues (Buss et al., 2015; Denyes et al., 2012). Contaminants such as heavy metals are intrinsic to some feedstocks, such as biosolids, and are neither created nor destroyed during pyrolysis (Chen et al., 2014; Zielińska & Oleszczuk, 2015). Through loss of volatiles from the feedstock, non-volatile heavy metals become more concentrated in biochar (Domene et al., 2015). Comparatively, PAHs are either native or generated during the pyrolysis process (Kambo & Dutta, 2015; Domene et al., 2015; Lievens et al., 2015; Wang, Wang & Herath, 2017). Heavy metals and PAHs are known toxicants to many organisms and hence could restrict the usage of derived biochars (Freddo, Cai & Reid, 2012; Domene et al., 2015).

Agricultural waste has been widely researched for biochar production (Zavalloni et al., 2011). Woody and herbaceous biomass presents advantages over other agricultural waste, as it can be harvested year-round, which eliminates long-term storage. In Victoria, Australia, agriculture produces annually >1.6 million dry tonnes of waste biomass as crop stubble, stems, kernels and grain processing residues (Victoria State Government, 2012a) and approximately 285,000 tons of timber wastes, including sawdust (Victoria State Government, 2012b), that could be beneficially converted to biochar materials. The generation of biochars from these wastes could potentially be an important tool for managing waste biomasses in an economical and sustainable manner. Furthermore, the State Government of Victoria has placed emphasis on the re-use of such biomass, as opposed to the practice of landfilling (Victoria State Government, 2012b).

Few authors have discussed the conversion products of woody and herbaceous biomasses at different pyrolysis temperatures. A growing number of studies are available which have characterized biochars derived from various waste streams as potential waste management and reuse strategies (Cely et al., 2015; Yargicoglu et al., 2015). However, there exist very few studies which compare the effects of production temperature and studied woody and herbaceous feedstocks (Srinivasan & Sarmah, 2015; Srinivasan et al., 2015) on resultant biochar characteristics at constant residence times. Table 1 contains comparative data for a small number of studies which have explored the characteristics to some extent for biochars derived from either pine (wood or sawdust) or straw. Table 1 demonstrates the current deficiency in biochar characterisation data for pine and straw feedstocks. It is also notable that there is a lack of information on trends specific to each feedstock with respects to the effect held by pyrolysis temperature on commonly measured parameters. Authors have noted the importance of such characterization studies for the optimization and designing of biochars in the future (Luo et al., 2015; Zhao et al., 2013).

**Table 1:** Literature values for biochars produced from pine or pea straw like feedstocks.

**Feedstock**	**T (°C)**	**t (min)**	**pH**	**SSA m**^**2**^**/g**	**FC (%)**	**VM (%)**	**Ash (%)**	**C (%)**	**H (%)**	**O (%)**	**N(%)**	**S(%)**	**Study and location**
Wheat (straw)	368	240	10.66	–	–	–	25.1	62.8	–	–	0.83	–	**Alburquerque et al. (2014) Spain**
Pine (woodchips)	428	228	8.38	–	–	–	4.4	80.0	–	–	0.37	–	
Pine (woodchips)	450	15	7.5	288	–	–	–	83.7	–	–	0.36	–	**Brennan et al. (2014)**
Pine(Wood)	350	60	–	28.7	71.8	–	2.63	–	–	–	–	–	**Das, Sarmah & Bhattacharyya (2016) New Zealand**
Pine(Wood)	420	10	–	0.7	69.7	–	2.06	–	–	–	–	–	
Pine(Wood)	470	10	–	0.9	74.5	–	1.81	–	–	–	–	–	
Pine(Wood)	900	60	–	335.9	82.2	–	13.4	–	–	–	–	–	
Pine (Sawdust)	300	60	–	8.2	–	–	4.58	55.3	5.50	39.0	0.07	0.13	**Luo et al. (2015) China**
Pine (Sawdust)	500	60	–	68.4	–	–	6.91	76.0	3.54	19.8	0.15	0.47	
Pine (woodchips)	450	15	7.5	–	–	–	1.8	85.2	2.78	–	0.37	–	**Moreno-Jiménez et al. (2018) Germany**
Pine (Sawdust)	680	10	9.7	795	–	–	1.01	90.9	1.31	0.11	6.1	–	**Srinivasan et al. (2015) New Zealand**
Wheat (Straw)	500	240	10.2	33.2	63.7	17.6	18	62.9	–	–	–	–	**Zhao et al. (2013) China**
Pine (Sawdust)	500	240	10.5	203	72.0	17.5	9.94	75.8	–	–	–	–	

**Notes.**

Where the following abbreviations or symbols are used – No data t Temperature, time SSA Specific Surface Area FC Fixed Carbon VM Volatile Matter C Carbon H Hydrogen O Oxygen N Nitrogen S Sulfur

In this study, six biochars were produced at three pyrolysis temperatures from two waste biomasses, pine sawdust (a softwood waste harvested all year) and pea straw (straw produced as an agricultural waste). These were chosen as they are common waste streams in Victoria, and each represents a biomass of differing structure and composition. Biochars were studied to assess the effect production temperature and feedstock specific composition had on each biochars unique characteristics. To our knowledge, this is the first study that has characterized a broad range of parameters and compared these two feedstocks and the effects pyrolysis temperature has on resultant biochars with increasing pyrolysis temperature at a constant residence time. Due to the temperature and feedstock specific nature of biochars, this work offers an important insight in the direction of “engineered biochars”.

## Materials and Methods

### Raw material selection

Six biochars were prepared using pine sawdust and pea straw as feedstocks. Sawdust was obtained from pine (*Pinus radiata*), grown in several plantations ranging between Eurobin (Victoria- 36°38′18.9″S 146°51′06.2″E) to Tumut (New South Wales, Australia - 35°18′58.6″S 148°13′51.7″E) plantations. Pea straw (*Pisum sativum*) was acquired from a wholesaler (Peninsula Hay) situated in the Mornington region of South East Victoria (38°24′12.9″S 144°58′34.6″E). In this region the pea plant is used to fix nitrogen in pastures and later harvested for use as feed or mulch.

### Pyrolysis of raw materials

Biochar was produced by tightly packing 400 g of a single feedstock into a 1 L internal volume (Radius–7 cm; Height–6.5 cm) stainless steel cylindrical vessel with a spring clamped lid which exerted a small downward force strong enough to prevent atmospheric exchange yet still allow evolved gases to escape under positive pressure. No inert gases were employed as oxygen was prevented from entering the vessel by the lid, any remaining oxygen existing in the vessel was either exhausted during heating or expelled through expansion during the temperature ramping process. Therefore, inside the vessel was considered an oxygen limited environment. The vessels were then placed in a furnace and the temperature ramped at 8.3 °C/min to a respective 350 °C, 500 °C or 750 °C, followed by a 1 h dwell time. These temperatures were selected as a gradient and are spread across the upper and lower as well as median thresholds for slow pyrolysis. After pyrolysis, each vessel was placed in the draft of a fume hood to allow an hour to cool before opening, to prevent ignition. The above process was carried out four times. All biochars were passed through a 1 mm sieve, homogenized and stored in polypropylene containers under standard lab conditions until analysis. Biochars were coded P (Pine Sawdust) and S (Pea Straw), and temperature groups (P350, P500, P750, S350, S500 and S750).

### Characterization of biochars

#### Chemical and physical characterization

Yield of the biochar was expressed as the percentage of biochar produced after pyrolysis relative to the initial mass of feedstock. Bulk density was calculated using the mass of biochar that could be packed into a 20 mL stainless steel cylinder with minimal compression (EBC, 2012). Proximate analysis was undertaken as per ASTM International, 2013; however, premature combustion of samples resulted in volatile matter (VM) requiring an alternate method. VM was measured using a Perkin Elmer Simultaneous Thermal Analyzer (STA) 6000, where 5 mg of sample was heated to 600 °C at a rate of 30 °C/min, in a nitrogen environment. Mass loss between 105 °C and 600 °C was considered the VM fraction. Fixed carbon (FC) was calculated as the remaining mass percentage after measured VM, ash and moisture percentages had been subtracted from the total mass.

Biochar pH and electrical conductivity (EC) were determined by preparing a 1:2 biochar:deionised water slurry (20 g biochar to 40 mL of water) then pH and EC determined in accordance with USEPA (2004). The alternate solid: liquid ratio was used to ensure wetting of entire biochar sample.

Surface area analysis was undertaken by N_2_ adsorption at 77 K using a Micromeritic ASAP 2400. Triplicate 10 mg samples were degassed at 100 °C for 8 h under low vacuum. Following no mass change after degassing, samples were degassed a further 12 h under high vacuum at 200 °C and was repeated until no mass change was evident. Biochars were fitted to a BET sorption isotherm to determine surface area.

A FEI Quanta 200 Scanning Electron Microscope (SEM) was used to examine surface morphology of biochars under low vacuum at 25 kV accelerating voltage, spot size of 6 nm, and at magnifications ranging 200–1, 600 × with a set working distance of 10.5.

Fourier Transform Infrared Spectroscopy (FTIR) analysis was carried out on a Perkin Elmer Spectrum 100 with single diamond/ZnSe attenuated total reflectance (ATR) module and pressure arm, as to delineate the dominant functional groups unique to each biochar.

Cation Exchange Capacity (CEC) was measured saturating the sample with a 0.5 M barium chloride solution then displacing the sorbed Ba^2+^ with a 1.0 M ammonium acetate solution (Mitchell et al., 2015). This extraction was employed to determine the sum of all cations (Mitchell et al., 2015) using inductively coupled plasma-optical emission spectrometry (ICP-OES).

Ultimate analysis was undertaken in accordance with International Organization for Standardization (2006) and International Organization for Standardization (2010). Oxygen content was calculated using ultimate analysis data by subtracting the sum of ash, carbon, nitrogen and hydrogen as a percentage from 100% (Enders & Lehmann, 2012). Biochar thermal stability was calculated as the percentage between fixed carbon divided by the sum of fixed carbon and VM. This calculated index value estimates the degree of thermal stability of each biochar, where values closer to one suggest a more stable biochar than those closer to zero (Alburquerque et al., 2013; Chen et al., 2014).

#### Contaminant analysis

Heavy metals (Cd, Cr, Cu, Pb, Hg, Ni, Zn), metalloid As and PAHs were analysed at an external certified commercial laboratory. Pseudo-total heavy metal analysis was undertaken using an adaptation of USEPA (1996), whereby heavy metals were extracted by refluxing of 0.1 g biochar samples in concentrated trace metals grade HNO_3_ and analysed by AAS and ICP-MS.

Sixteen priority USEPA PAHs, 7,12-Dimethylbenz(a)anthracene and 3-Methylcholanthrene were determined by ultrasonic extraction of samples which were quantified by gas chromatography–mass spectrometry (GC/MS) in accordance with USEPA (1998). The values of heavy metals and PAHs were compared with the limits stipulated in the *Standardized Product Definition and Product testing Guidelines For Biochar That is Used In Soil* (International Biochar Initiative, 2013).

### Statistics

Descriptive statistics, Two-way factorial ANOVA and Pearson Correlation were carried out on IBM’s SPSS Statistics 22 package (Armonk, NY, USA). Two- way ANOVA was the key tool in verifying significant trends between biochar parameters governed by pyrolysis temperature and differences between feedstock type. Univariate factorial analysis allowed the identification of the main effect responsible for any trends observed, differentiated as temperature, feedstock or interaction. Significant results are displayed in the format (*F*_1,6_ = X, *p* < 0.05), where the *p* value is alongside the F value. F-crit values can be ascertained using F tables and the subscript numbers, the first being the degrees of freedom followed by the number of sample groups for that parameter. Pearson Correlation results are displayed as follows (R = X, n = X, p = X), where *R* is the correlation factor, *n* denotes the number of groups of samples sampled and the last figure corresponds to the *p* value.

## Results and Discussion

### Chemical and physical characterization

Temperature was found to be the main effect influencing yield (*F*_2,18_ = 12.1, *p* < 0.05), with yields decreasing at higher pyrolysis temperatures for both, straw and pine feedstocks. Straw exhibited greater yields than pine at both 500 °C and 750 °C (25% and 23% compared to 21% and 20% respectively), while at 350 °C pine (34%) produced a higher yield than straw (29%). This is likely due to the interplay of temperature and the two feedstocks, which differ in water, lignin, cellulose and hemicellulose composition. Feedstock and temperature have been identified as the most influential parameters in the decomposition of woody and herbaceous biomasses to produce biochar (Zhao et al., 2013; Benavente et al., 2018). This is due to the variation in each feedstock with respect to their content of the biopolymers; hemicellulose, cellulose and lignin, all of which degrade at different temperature ranges (Qian et al., 2015; Kambo & Dutta, 2015; Yeo et al., 2017). Comparatively the rigid structure required by trees results in a higher proportion of lignin in softwoods than in herbaceous grasses, while grasses are more cellulosic (Das & Sarmah, 2015; Azargohar et al., 2013). Hemicellulose is the easiest degraded of the three major components, with complete degradation starting at 330 °C (Yeo et al., 2017; Buss et al., 2015). The greatest proportion of cellulose degradation occurs at temperatures above 427 °C, though degradation can begin at lower temperatures, generating much volatile matter as carbon-oxygen and carbon-hydrogen bonds are broken (Buss et al., 2015). Lignin is the most recalcitrant of these three major components, with complete degradation evident only after temperatures exceeding 607 °C. This is due to lignin’s structure consisting of multiple ether linkages and functional groups such as hydroxyl and methoxy (Yeo et al., 2017).

**Figure 1:**
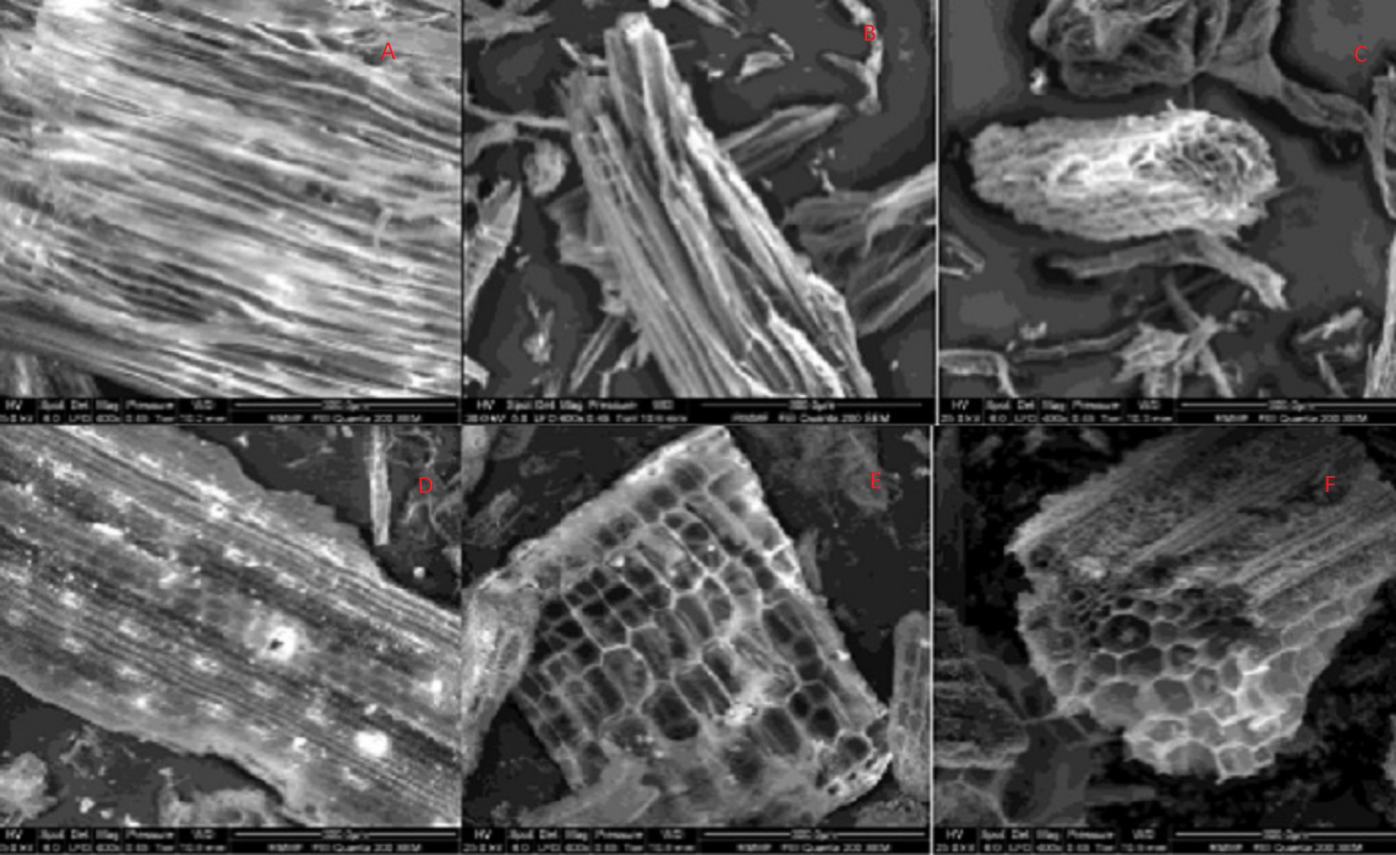
SEM Image of biochar surfaces derived from pine pyrolyzed at 350 °C, 500 °C and 750 °C (A, B and C, respectively) and straw pyrolyzed at 350 °C, 500 °C and 750 °C (D, E and F, respectively). All images obtained at 400 × magnification.

A difference in surface morphology was observed using SEM in the form of cellular structure between the feedstocks, as well as, increased fracturing of structure with increased pyrolysis temperature (Fig. 1). The cells seen in pine biochars were longer and more cylindrical than the short cuboid cells noted in straw based biochars, and the pores visible on the surface of all biochars were similar to those reported in previous literature (Shaaban et al., 2014).

Bulk density was similar across feedstocks, ranging between 0.12–0.17 g/cm^3^. Pine at 350 °C had the lowest bulk density, however at 750 °C both biochar types were matched in density (Table 2). This would have implications when biochars are used as soil conditioner. Thus, bulk density is of utmost importance to rainfall infiltration. Moreover, a decrease in bulk density would have ramifications, increasing soil porosity and soil aeration, and, potentially leading to a positive effect on microbial respiration.

**Table 2:** Summary of characteristics determined for biochars produced in study.

Biochar	P350	P500	P750	S350	S500	S750
**Biochar physiochemical characteristics**						
Surface Area (m^**2**^/g)	16.2 ± 5.2^A,c^	278.0 ± 4.7^A,b^	397.4 ± 4.1^A,a^	22.2 ± 3.6^B,c^	46.7 ± 8.9^B,b^	157.7 ± 4.2^B,a^
Moisture (%)	1.54 ± 0.01^A,c^	1.73 ± 0.13^A,b^	3.29 ± 0.06^A,a^	1.72 ± 0.01^B,b^	1.76 ± 0.03^A,b^	4.01 ± 0.04^B,a^
Volatile Matter (%)	15.29 ± 0.59^A,a^	4.28 ± 0.16^B,b^	2.88 ± 0.08^B,c^	16.23 ± 1.15^A,a^	14.49 ± 0.42^A,a^	7.50 ± 0.32^A,b^
Ash (%)	1.16 ± 0.01^B,b^	1.88 ± 0.01^B,a^	1.92 ± 0.06^B,a^	15.24 ± 0.17^A,b^	16.21 ± 1.67b^A,b^	23.95 ± 0.29^A,a^
Fixed Carbon (%)	82.01 ± 2.02^A,b^	92.11 ± 1.97^A,a^	91.91 ± 3.07^A,a^	66.81 ± 3.18^B,a^	67.54 ± 2.84^B,a^	64.54 ± 2.98^B,b^
Bulk Density (g/cm^**3**^)	0.12 ± 0.01^B,c^	0.15 ± 0.01^A,b^	0.17 ± 0.02^A,a^	0.15 ± 0.01^A,c^	0.16 ± 0.01^A,b^	0.17 ± 0.02^A,a^
Thermal Stability	0.84 ± 0.10^A,c^	0.96 ± 0.04^A,b^	0.97 ± 0.04^A,a^	0.81 ± 0.16^A,b^	0.82 ± 0.08^B,b^	0.90 ± 0.05^B,a^
**Biochar ultimate analysis**						
Carbon (%)	75.6 ± 2.1^A,c^	88.0 ± 1.3^A,b^	93.8 ± 0.9^A,a^	61.3 ± 2.7^B,b^	64.4 ± 2.5^B,a^	63.9 ± 2.7^B,a^
Sulphur (%)	0.07 ± 0.01^B,a^	0.07 ± 0.01^B,a^	0.08 ± 0.01^B,a^	0.27 ± 0.02^A,a^	0.27 ± 0.02^A,a^	0.21 ± 0.01^A,b^
Nitrogen (%)	0.25 ± 0.01^B,c^	0.41 ± 0.03^B,b^	0.56 ± 0.01^B,a^	1.08 ± 0.11^A,a^	1.11 ± 0.09^A,a^	0.95 ± 0.13^A,a^
Hydrogen (%)	4.73 ± 0.27^A,a^	3.08 ± 0.15^A,b^	1.07 ± 0.18^A,c^	3.89 ± 0.14^B,a^	2.52 ± 0.12^B,b^	0.66 ± 0.07^B,c^
Oxygen (%)	18.26 ± 2.12^A,a^	6.63 ± 1.31^B,b^	2.65 ± 0.92^B,c^	18.50 ± 2.71^A,a^	15.76 ± 3.01^A,b^	10.54 ± 2.72^A,c^
Inorganic (%)	1.09 ± 0.08^A,b^	1.81 ± 0.11^A,a^	1.84 ± 0.09^A,a^	14.96 ± 0.79^B,c^	15.94 ± 2.18^B,b^	23.74 ± 2.24^B,a^
H:C	0.06 ± 0.01^A,a^	0.04 ± 0.01^A,b^	0.01 ± 0.01^A,c^	0.06 ± 0.01^A,a^	0.04 ± 0.01^A,b^	0.01 ± 0.01^A,c^
C:N	302.40 ± 121.10^A,a^	214.63 ± 90.71^A,b^	167.50 ± 29.96^A,c^	56.76 ± 17.49^B,a^	58.02 ± 15.75^B,a^	67.26 ± 25.59^B,b^
O:C	0.24 ± 0.12^B,a^	0.08 ± 0.01^B,b^	0.03 ± 0.01^B,c^	0.30 ± 0.03^A,a^	0.24 ± 0.03^A,b^	0.17 ± 0.03^A,c^

**Notes.**

Statistically significant relationships (*P* < 0.05) are denoted in table by capital letters (A, B) for feedstock and lowercase letters (a,b,c) for temperature. Values are presented as mean ± standard deviation.

Surface area increased in both feedstock types with higher pyrolysis temperature and large surface area differences were observed between feedstock types (Table 2). Feedstock (*F*_1,11_ = 529.0, *p* < 0.05), temperature (*F*_2,11_ = 471.6, *p* < 0.05) and their interactions (*F*_2,11_ = 132.5, *p* < 0.05) were significant factors with respects to biochar surface area and this is consistent with similar studies, carried out in other lignocellulosic wastes (Das & Sarmah, 2015). Pine biochars had higher surface areas than straw biochars in the 500 °C (278.0 ± 4. cm^2^/g) and 750 °C (397.3 ± 4.1 cm^2^/g) experiments, however the depressed values at 350 °C (16.3 ± 5.2 cm^2^/g) were suggested to be due to the lower temperatures resulting in the underdevelopment of pores (Abdel-Fattah et al., 2015) and clogging of pores with tars which could not volatilize (Das & Sarmah, 2015). These results fit within the range expressed by in Table 1 for surface area.

Moisture levels ranged between 1.5 and 4.0% in biochars produced, an increase in moisture was observed with higher pyrolysis temperature (Table 2). Temperature and feedstock were each found to hold significant effects over biochar moisture levels (*F*_1,6_ = 9.6, *p* < 0.05 and *F*_1,6_ = 169.4, *p* < 0.05, respectively). Biochars prepared at 750 °C had the highest moisture levels, particularly P750, it is suggested that this is absorbed from the atmosphere due to the higher surface area of the material.

Temperature was found to be a main effect for VM content (*F*_2,6_ = 42.9, *p* < 0.05), decreasing from 350 °C biochars to 750 °C biochars (Table 2). This is consistent with other studies (Table 1) as VM loss occurs as outgassing volatiles (Al-Wabel et al., 2013). Feedstock was also identified as a main effect (*F*_1,6_ = 31.4, *p* < 0.05), exhibiting higher average VM in straw biochars than pine biochars. An interaction for both factors was present *F*_2,6_ = 8.3, *p* < 0.05 suggesting an interplay between these two factors. Volatile matter is of importance to explain the microbial and plant responses following biochar addition to the soil, although this is a poorly understood interaction, due to the large amount of individual volatile compounds present in biochars (Spokas et al., 2011).

For ash, feedstock was determined to be a main effect (*F*_1,6_ = 219.7, *p* < 0.05), such that ash content was significantly higher in straw biochar than pine. Ash fractions increased with higher temperature (Table 2), which was demonstrated to be a main effect by univariate factorial ANOVA (*F*_2,6_ = 6.6, *p* < 0.05). This demonstrates temperature’s role in forming the ash fraction, compared to feedstocks role in defining the fraction available for maximum ash formation. Further, a statistically significant interaction between feedstock and temperature was found (*F*_2,6_ = 5.3, *p* < 0.05). Ash represents the largely inorganic fraction that cannot be volatized or degraded by combustion, including potassium (K), calcium (Ca) magnesium (Mg), carbonates and heavy metals (Hmid et al., 2014). Ash compounds hinder the formation of aromatic structures that contribute greatly to fixed carbon content.

Fixed carbon was found to be higher in pine biochars than in straw biochars (Table 2). Feedstock was found to be the main effect for this difference in fixed carbon between biochars (*F*_1,2_ = 36.9, *p* < 0.05) and is supported by literature (Zhao et al., 2013). FC values were slightly higher than those seen in literature (Table 1). Fixed carbon values were in agreement with those of the thermostability index. In general, higher values of these would be indicative of a longer residence time of biochar in soil.

Thermal stability increased with pyrolysis temperature for all biochars, lower thermal stability was noted for straw biochars (Table 2). This suggests that pine biochars will be more recalcitrant in the environment than straw biochars (Das, Sarmah & Bhattacharyya, 2016).

**Figure 2:**
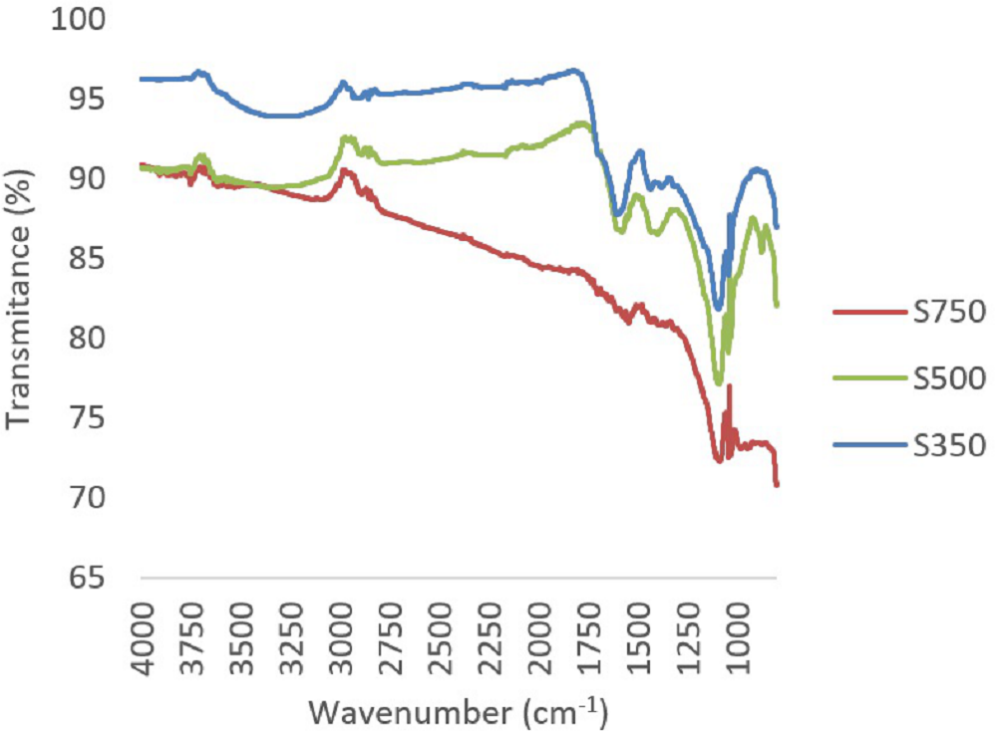
Composite FTIR spectra of biochars produced from straw at 350 °C, 500 °C and 750 °C.

**Figure 3:**
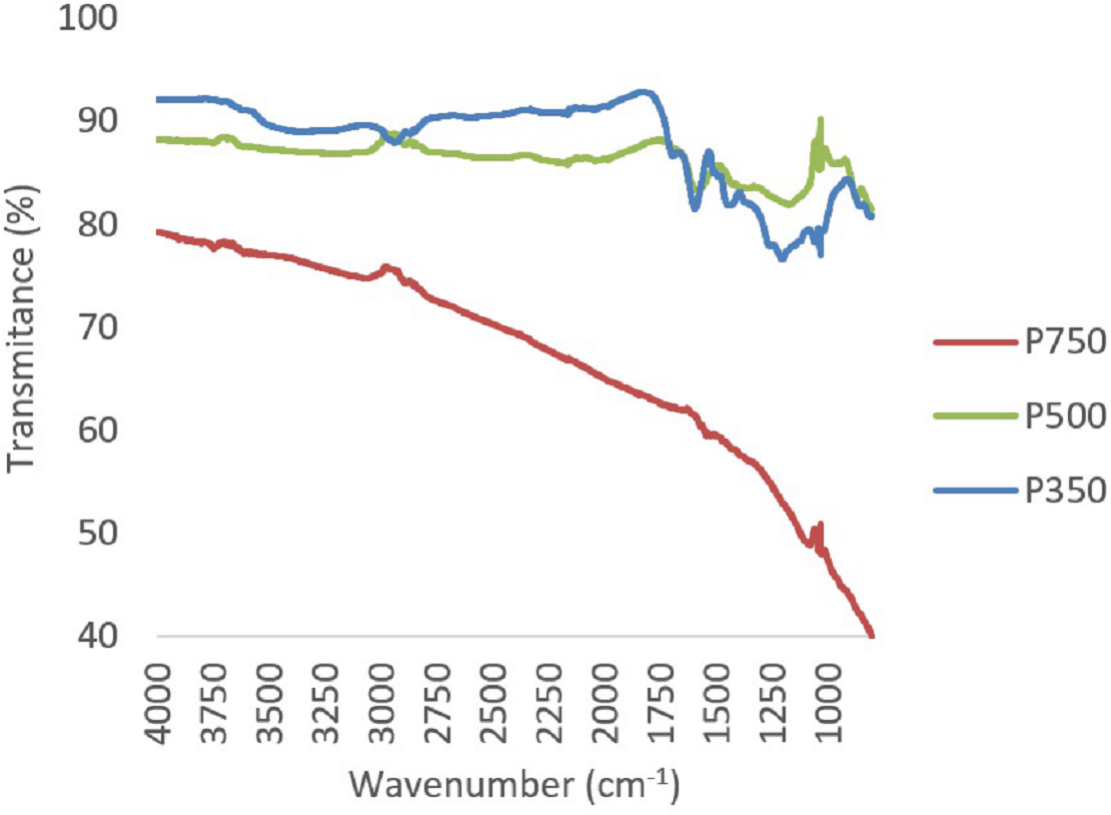
Composite FTIR spectra of biochars produced from pine at 350 °C, 500 °C and 750 °C.

A reduction in oxygen and hydrogen containing functional groups, primarily carbonyl (1,690–1,700 cm^−1^) and carboxyl groups (1,690–1,760 and 1,210–1,320 cm^−1^) between 350 °C and 500 °C, was observed through FTIR (Figs. 2 and 3). In both feedstock types, FTIR suggests that at 750 °C biochars are namely comprised of C-C bonds and that most of the other functional groups and volatile components had been lost, this is similar to trends expressed in by results found for other biochars produced from woody feedstocks (Alburquerque et al., 2013; Abdel-Fattah et al., 2015). In all biochars, H and O values were comparable to literature, though slightly lower values (Table 2). Hydrogen content decreased with higher pyrolysis temperature and a difference was observed between feedstocks, with hydrogen in straw derived biochars being lower than in pine biochars (Table 2). Both feedstock and temperature significantly influenced hydrogen content (*F*_2,2_ = 250.4, *p* < 0.05 and *F*_1,2_ = 22.9, *p* < 0.05 respectively).

Nitrogen was not affected by higher pyrolysis temperature in straw biochars, however N increased with pyrolysis temperature for pine biochars (Table 2). Feedstock was the main factor affecting nitrogen content (*F*_1,2_ = 24.0, *p* < 0.05). Similarly, feedstock was the main factor influencing percentage sulphur remaining in biochars (*F*_1,2_ = 57.3, *p* < 0.05). Sulphur and nitrogen values for all biochars compared well to literature values seen in Table 1.

H:C ratio is a measure often used to discern the degree of aromatization in biochars as increases in carbon are inversely related to hydrogen through polymerization, dehydration and volatization. In these experiments H:C decreased in both biochar types with higher pyrolysis temperature, highlighting temperature as a main effect (*F*_2,2_ = 393.1, *p* < 0.05) and suggesting an increase in aromatization (Table 2). The loss of H is indicative of water and surface acid functional group loss, such as hydroxyl (OH) and carboxyl (COOH) through volatilization. Higher pyrolysis temperatures results in a greater loss of VM, oxygen and hydrogen as due to depolymerisation of biopolymers and the carbonisation of the feedstock to a more recalcitrant form through decarboxylation, dehydration, de-carbonylation, de-methylation, condensation and aromatisation reactions (Das & Sarmah, 2015; Heitkötter & Marschner, 2015).

Inverse relationships were observed between moisture and H:C (*R* =  − 0.912, *n* = 6, *p* = 0.006); bulk density and H:C (*R* =  − 0.827, *n* = 6, *p* = 0.021); and bulk density and yield (*R* =  − 0.851, *n* = 6, *p* = 0.016). The relationships between bulk density, yield, and H:C can all be understood through mass loss. Hydrogen is lost through dehydration while percentage carbon content increases through condensation and graphitization, affecting H:C. Yield decreases in proportion to H:C via mass loss, whereas bulk density increases due to the formation of graphite like structures. These are temperature dependent relationships, though the initial feedstock does play a major role in their resilience to thermal degradation. It is intuitive that moisture levels decrease from feedstock to biochar through the loss of water as steam due to the elevated temperatures used in pyrolysis. However, the increase in moisture in finished biochars as a function of temperature is surmised to be due to the hygroscopic effect exerted by their high surface area. This is further supported by the correlations seen in BET surface area relating in a negative manner to yield (*R* =  − 0.824, *n* = 6, *p* = 0.022), O: C (*R* =  − 0.976, *n* = 6, *p* < 0.001) and VM (*R* =  − 0.964, *n* = 6, *p* = 0.001), all of which are characteristics which decrease with higher pyrolysis temperature. This relationship highlights the impact outgassing of VM has in the formation of pores and hence a higher surface area (Das & Sarmah, 2015).

**Figure 4:**
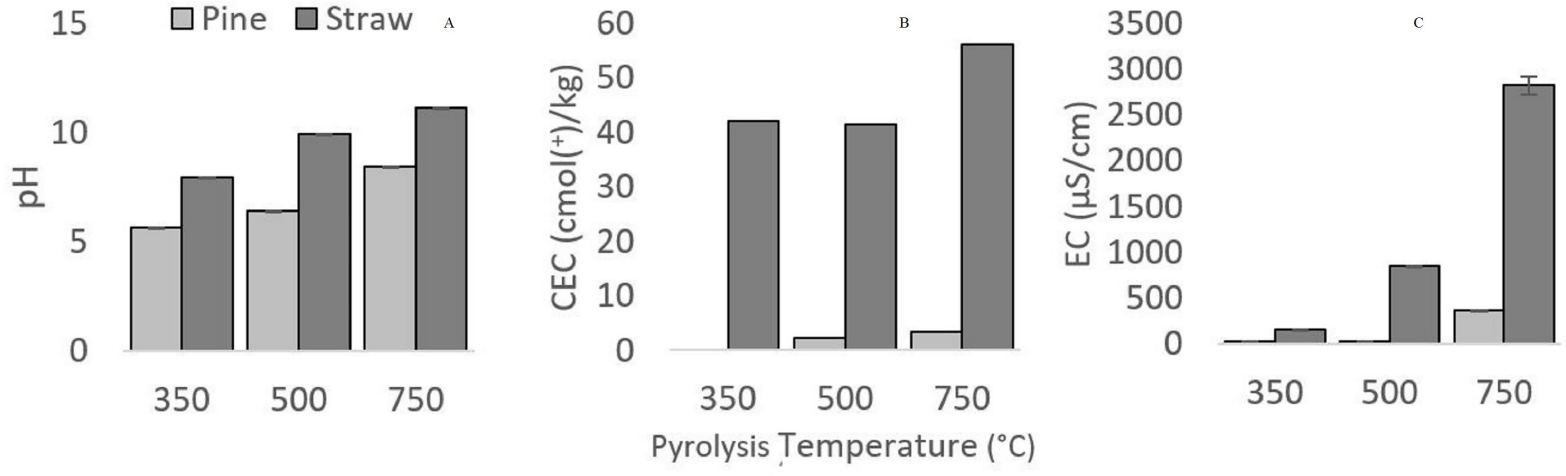
pH, EC and CEC of biochars, compared between feedstocks (Pine and Straw) and pyrolysis temperatures (350, 500 and 750 °C). pH (A), EC (B) and CEC (C)

Electrical conductivity (EC) and pH increased with higher pyrolysis temperature in all cases. Both pH and EC were found to be higher in straw biochars than in pine biochars (Fig. 4). Temperature (pH: *F*_2,6_ = 1706.8, *p* < 0.05; and EC: *F*_2,6_ = 179.5, *p* < 0.05), feedstock (pH: *F*_1,6_ = 4621.7, *p* < 0.05; and EC: *F*_1,6_ = 279.4, *p* < 0.05) and their interactions (pH: *F*_2,6_ = 73.5, *p* < 0.05; and EC: *F*_2,6_ = 103.0, *p* < 0.05) were found to play a significant role in pH and EC values. This suggests the differences within feedstock groups were due to pyrolysis temperature, the different values across temperature groups were due to feedstock and the extent of the difference was due to the interaction of these two factors. Increases in EC are the result of a gain in the amount of ions present through the increase in ash fraction (Alburquerque et al., 2013). Similarly, it is well-established that increasing pyrolysis temperature tends to favour the alkalinity of biochars (Yuan, Xu & Zhang, 2011). This is partly due to an increase in inorganic carbonates (Yuan, Xu & Zhang, 2011).

CEC denotes the ability of biochar to bind cations (Hmid et al., 2014), and was found to increase with higher pyrolysis temperatures and was higher in straw derived biochars (Fig. 4). Feedstock was the main factor influencing the differences between biochars (*F*_1,2_ = 115.9, *p* < 0.05).

Strong positive Pearson correlations were observed between pH, ash, and CEC creating a grouping of correlated parameters (*p* < 0.05). These correlations were in line with expected increases caused by pyrolysis temperature and therefore increased ash content, which result in increased pH through the concentration of ions such as K, Ca, Mg and carbonates (Kuzyakov, Bogomolova & Glaser, 2014; Heitkötter & Marschner, 2015). These increases in pH elevated the biochars CEC as this parameter is pH dependent (Abdel-Fattah et al., 2015). In addition, significant positive correlations regarding sulphur (*R* = 0.858, *n* = 6, *p* = 0.014) and nitrogen (*R* = 0.868, *N* = 6, *p* = 0.013) with ash were obtained.

### Contaminant analysis

Six of the eight heavy metals tested were detected in the biochars, with mercury and cadmium being below the limit of quantitation (<LQ) (2mg/kg) in all samples (Table 3). Lead, nickel and zinc were found to be below IBI guidelines in all biochars as illustrated by (Table 3). Similarly, arsenic, chromium and copper were below guideline levels in biochars produced from straw feedstocks.

**Table 3:** Heavy Metal concentrations detected in biochars (mg/kg).

Biochar	P350	P500	P750	S350	S500	S750	IBI Limits
Arsenic	1,400 ± 88^a^	410 ± 17^b^	190 ± 19^c^	<LQ	<LQ	<LQ	13–100
Cadmium	<LQ	<LQ	<LQ	<LQ	<LQ	<LQ	1.4–39
Chromium	1,400 ± 21^A,a^	180 ± 19^A,b^	13 ± 3^A,c^	2 ± 1^B,c^	3 ± 1^B,b^	13 ± 2^A,a^	93–1,200
Copper	900 ± 14^A,a^	880 ± 17^A,a^	650 ± 17^A,b^	15 ± 2^B,c^	20 ± 2^B,b^	85 ± 6^B,a^	143–6,000
Lead	2 ± 1^A,a^	2 ± 1^A,a^	2 ± 1^B,a^	5 ± 1^A,b^	3 ± 1^A,b^	26 ± 7^A,a^	121–300
Mercury	<LQ	<LQ	<LQ	<LQ	<LQ	<LQ	1–17
Nickel	<LQ	<LQ	<LQ	<LQ	2 ± 1^a^	5 ± 1^b^	47–420
Zinc	11 ± 1^A,a^	10 ± 1^A,a^	13 ± 1^A,a^	39 ± 1^B,c^	29 ± 2^B,b^	120 ± 5^B,a^	416–7,400

**Notes.**

IBI Guidelines heavy metals in biochars are presented as an interval as per the original source, due to the different soil tolerance level for these elements in regulatory bodies in the US, Canada, EU and Australia. <LQ represents data points at which all samples were below the limit of quantification reporting value (2 mg/kg). Statistically significant relationships (*P* < 0.05) are denoted by capital letters (A, B) for feedstock and lowercase letters (a, b, c) for temperature. Values are presented as average values ± standard deviation.

Biochars produced from pine had elevated levels of arsenic, copper and chromium that were 1–2 orders of magnitude above IBI limits in all pine biochars excepting chromium at 750 °C. The presence of these three heavy metals at elevated concentrations was due to raw pine being milled alongside treated pine, in turn contaminating the feedstock with chromated copper arsenate. Chromated copper arsenate is the most commonly used wood preservative and treated woods typically contain chromium, copper and arsenic concentrations within the range 2.6–9.8 mg g^−1^, 5.3–19.0 mg g^−1^ and 5.2–16.3 mg g^−1^ respectively (Jones & Quilliam, 2014). It is likely, in Victoria and elsewhere, that timber from construction waste or wood sourced from forests, would have significant concentrations of chromated copper arsenate, which would impact the quality of the biochar. If these heavy metals are allowed to enter into the environment, through biochar application, their excessive availability could have detrimental toxic effects on local wildlife or crops (Alburquerque et al., 2013; Freddo, Cai & Reid, 2012). Typically, metals measured and detected increase in concentration with higher pyrolysis temperature as found in previous work (Benavente et al., 2018). However, there are exceptions. It is likely that arsenic could be lost as volatile arsenic trioxide (As_2_O_3_) during pyrolysis ((Jones & Quilliam, 2014); (Helsen & Van den Bulck, 2000) as it has been demonstrated that 15–24% of arsenic concentration can be lost during incineration at 400 –800 °C (Yan et al., 2008). While it is possible for a fraction of arsenic to be liberated as the volatile arsenic trioxide in the presence of oxygen, it does not account for the magnitude of arsenic loss seen in this study within an oxygen limited environment at high temperature. A dissimilar trend with temperature was found for copper and chromate concentrations in pine and straw biochars. This result could be due to differences in the partition of heavy metals between the biochar and the tar. Differences in the molecular structure of the feedstocks and how contaminants are distributed in the matrix could have contributed to these results (Farrell, Rangott & Krull, 2013). Further, it has been demonstrated that with respects to total heavy metals the result can be dependent on the extraction method (Enders & Lehmann, 2012). Hence the term pseudo-total heavy metals must be applied, as this designates that it is the maximum that can be extracted for the method used, which is often determined by availability of lab equipment and access to materials and reagents (Beesley, Moreno-Jiménez & Gomez-Eyles, 2010). In this study the remainder of the heavy metals tended to be higher in concentration in straw derived biochars than in pine. Levels for Zn were comparable to those seen for similar feedstocks for both straw and pine biochars, the same is true of Pb in pine biochars (Srinivasan & Sarmah, 2015; Zhao et al., 2013), demonstrating the small pool of analogue studies from which to draw literature comparisons in this area. In Australia or in the state of Victoria, there are currently no specific biochar application guidelines and it is necessary to rely on IBI guidelines for heavy metal limits (Table 3). Using these criteria all straw biochars qualify as soil enhancers and all pine biochars would be unsuitable for land application due to elevated arsenic concentrations (International Biochar Initiative, 2013). It has been demonstrated that elevated levels of arsenic, chromium and copper have a negligible effect on the determination of other parameters such as EC and pH (Jones & Quilliam, 2014). Elevated metals should not interfere with ultimate analysis, proximate analysis or surface area determination.

The sixteen USEPA PAHs measured in the study were below the limit of detection (0.5 mg/kg) in most biochars with the exceptions being S500 and P350. S500 was found to contain a single PAH, 1.2 mg/kg naphthalene. Comparatively, P350 contained a total of 6 PAHs (Table 4), with 7,12-dimethylbenz(a)anthracene concentration dominating at 35.0 mg/kg. . Out of the 6 biochars created, the PAH level in one biochar (P350) is above the suggested IBI PAH limit (6 mg/kg) (International Biochar Initiative, 2013). PAHs are generated during biochar production through incomplete combustion of biomass. PAH concentration in biochars is feedstock-dependent (Wang, Wang & Herath, 2017). Naphtalene is usually the major hazardous PAH (Wang, Wang & Herath, 2017). In agreement with our results, it is well documented that PAH concentration in biochars diminishes with the temperature of pyrolysis (Wang et al., 2018). Oleszczuk, Jośko & Kuśmierz (2013) demonstrated that in environmentally relevant applications to soil (10% rate of addition), biochar’s PAH content could inhibit root growth for *Lepidium sativum* up to 92% compared to controls. Root growth inhibition started at concentrations of 5%. Further a significant relationship was established between total PAHs, leached from biochars using water, and the mortality of a test planktonic crustacean (Daphnia magna). These demonstrate the threat biochars containing PAHs could pose to the environment and agricultural lands if application rates appropriate to each biochar are not determined.

**Table 4:** PAHs concentration in biochars (mg/kg).

Biochar	P350	P500	P750	S350	S500	S750
Benzo(k)Fluoranthene	**0.9**	<LQ	<LQ	<LQ	<LQ	<LQ
7,12- Dimethylbenz(a)anthracene	**35.0**	<LQ	<LQ	<LQ	<LQ	<LQ
Fluoranthene	**0.9**	<LQ	<LQ	<LQ	<LQ	<LQ
Naphthalene	**0.6**	<LQ	<LQ	<LQ	**1.2**	<LQ
Phenanthalene	**0.6**	<LQ	<LQ	<LQ	<LQ	<LQ

**Notes.**

Data only displayed for PAHs with values above detection limit in at least one biochar. IBI Guideline maximum accumulative USEPA 16 PAH concentration 6 mg/kg. “<LQ” represents data points at which all samples were below the limit of quantification (<0.2 mg/kg).

## Conclusion

Six biochars of varying physiochemical properties were successfully engineered through slow pyrolysis at three selected temperatures, using waste feedstocks common in Victoria, Australia. It was found that both temperature and feedstock type were influential on the types of biochars created from selected biomasses and could in turn determine their suitability for environmental application.

All straw biochars created were compliant with IBI guidelines, with respects to contaminant burden. Pine biochars contained excessive levels of arsenic, chromium and copper due to contamination with a common wood treating agent, chromated copper arsenate. Low temperature pine biochar (350 °C) contained a number of PAHs exceeding both accumulative and individual limits, rendering them unsuited to land application; however PAHs could be eliminated by employing higher pyrolysis temperatures.

A range of different biochars with varying carbonized fractions, surface areas and functional groups were produced. The biochars created generally exhibited characteristics favourable for soil enhancement, such as elevated fixed carbon, CEC, pH and low bulk density. Our results can assist in decision making for biochars which could be engineered from pine sawdust and straw waste biomasses for specific environmental amelioration purposes, while aiding in reducing the amount of biomass reaching landfill and reducing carbon emissions.

##  Supplemental Information

10.7717/peerj.6784/supp-1 Data S1 Raw data for the characteristics of the biochars

## Additional Information and Declarations

### Data Availability

The following information was supplied regarding data availability: All average data and standard deviation values are included in the article. Raw data are available in Data S1.
